# Influence of the volume of restorative material on the concentration of stresses in the restorative interface

**DOI:** 10.4317/jced.57781

**Published:** 2021-06-01

**Authors:** Marina Pace, Josué-Junior Pierote, João-Victor Câmara, Isabel Barbosa, Cíntia-Tereza Araújo, Lucia Prieto, Guereth-Alexsanderson Carvalho, Gisele Pereira, Renato Vianna, Hana Fried, Justine Tinoco, Amara Santos, Luis-Alexandre Paulillo

**Affiliations:** 1 DDS, Department of Restorative Dentistry, Piracicaba Dental School, State University of Campinas, Piracicaba, São Paulo, Brazil; 2 Postdoctoral student, Department of Restorative Dentistry, Piracicaba Dental School, State University of Campinas, Piracicaba, São Paulo, Brazil; 3 Master student, Department of Biological Sciences, Bauru School of Dentistry, University of São Paulo, Bauru, São Paulo, Brazil; 4 PhD, Department of Restorative Dentistry, Piracicaba Dental School, State University of Campinas, Piracicaba, São Paulo, Brazil; 5 Adjunct Professor, Department of Dentistry, Faculty of Health Sciences, Federal University of Jequitinhonha and Mucuri Valleys, Diamantina, Minas Gerais, Brazil; 6 Master student, Department of Dentistry, Federal University of Piauí, Teresina, Piauí, Brazil; 7 Adjunct Professor, School of Dentistry, Federal University of Rio de Janeiro, Rio de Janeiro, RJ, Brazil; 8 School of Dentistry, Federal University of Rio de Janeiro, Rio de Janeiro, RJ, Brazil; 9 Titular Professor, Department of Restorative Dentistry, Piracicaba Dental School, State University of Campinas, Piracicaba, São Paulo, Brazil

## Abstract

**Background:**

To evaluate the microtensile strength in the adhesive interface depending on the volume of the composite resin used to restore class I cavities.

**Material and Methods:**

Forty-eight human third molars received a standardized class I cavity preparation and they were separated into six experimental groups: G1 – single-bottle adhesive system; G2 - bonding system with load; G3 – single-bottle adhesive associated with low-viscosity composite resin; G4 – loaded adhesive associated with low-viscosity composite; G5 - resin-modified glass ionomer associated with single-bottle adhesive; and G6 - resin-modified glass ionomer associated with loaded adhesive. All cavities were restored with a universal restorative composite. After completing the restorations, the samples were stored for seven days in a stove (37°C) and the microtensile bond strength was evaluated by producing slices and applying axial loading in an Instron universal testing machine at a speed of 0.5 mm/min. The thickness of the intermediate layer formed on the cavity floor to verify the relationship between the volume of restorative composite and the concentration of stresses in the buccal wall. With the data obtained in the microtensile strength test, an Analysis of Variance (ANOVA) was performed entirely at random.

**Results:**

Duncan’s test showed that group 4 (Filtek Flow/Optibond Solo Plus) obtained the highest mean of microtensile strength with no statistically significant difference to groups 3 (Filtek Flow/Single Bond), 5 (Vitremer/Single Bond), and 6 (Vitremer/Optibond Solo Plus). It also showed a statistically significant difference to groups 2 (Optibond Solo Plus) and 1 (Single Bond), with no statistical difference between the other groups studied.

**Conclusions:**

The highest mean of microtensile strength was obtained when the volume of the restorative material decreased through the interposition between the material and the adhesive system of a base with low elasticity modulus.

** Key words:**Adhesion, microtensile, composite resin.

## Introduction

Polymerization shrinkage is an intrinsic characteristic of composite resins that causes clinical inconveniences such as postoperative sensitivity, discoloration, and marginal cracks, and it may consequently lead to the development of recurrent caries (1).

The clinical problems from polymerization shrinkage increase when the composites are used to restore posterior teeth. This is probably due to the high configuration factor (C-factor) found in these cavities. The C-factor is the ratio between bonded and free surfaces of the cavity preparation, and the higher this ratio, the less freedom the material has to dissipate stresses during polymerization shrinkage (2).

Another aspect related to the clinical problems caused by composite resin shrinkage in posterior teeth is the volume of restorative material used. The higher the volume of composite inserted in the cavity, the higher the volumetric shrinkage of the restoration after polymerization (3,4). Even if the composite is inserted in small increments or oblique increments, the clinical inconveniences caused by the concentration of stresses in the restorative interface may be observed (5).

Considering the different clinical possibilities to reduce the stresses produced by composite resin shrinkage in the restorative interface in high C-factor cavities, it is important to evaluate the influence of reducing the volume of composite through the interposition of different base or coating materials. Thus, this study aimed to evaluate the microtensile strength in the adhesive interface depending on the volume of composite resin used to restore class I cavities.

## Material and Methods

-Obtaining and preparing the samples

Forty-two healthy human third molars were obtained with similar sizes and closed root apices and stored in 0.9% thymol saline solution at a temperature of 5°C (Arias, 2003), for a maximum of 30 days.9 The teeth were obtained through formal and informed donations, according to the regulation of the Research Ethics Committee of the School of Dentistry of Piracicaba (SP, Brazil).

After scraping the external surface with periodontal instruments and cleaning with sodium bicarbonate and water blasts (Profi II- Dabi Atlante Ltda.), the mesiodistal and bucco-palatal diameters were measured using a digital caliper (Digimess Ind. e Com. Ltda.) so that teeth with approximate sizes would be used in the study. Using a magnifying glass with a magnification of four times, the teeth were assessed for the presence of cracks or other superficial defects. Teeth that showed great variations in size or structural defects were discarded and new third molars were obtained.

-Materials

To carry out the study, the materials used were: Single Bond, Vitremer, Optibond Solo Plus and Filtek Flow. The classification, composition and manufacturer are described in Table 1.

**Table 1:**
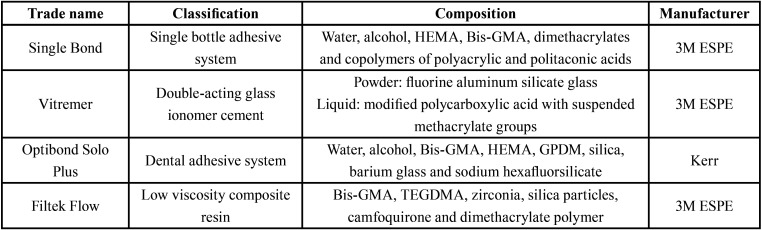
Trade name, classification, composition and materials manufacturers used in the restoration of class I cavities.

-Inclusion of teeth

To stabilize the teeth so they would be later fixed in the cavity preparation machine, they were positioned perpendicularly to the horizontal plane on #7 pink wax plates through the root apex, and PVC rings (Tigre S. A. Tubos e Conexões - Ind. Bras.) with 5 mm of height and 12.7 mm of internal diameter were positioned around the teeth, so they would be internally centralized. The margins were sealed with the thermoplasticization of the wax with a 7 spatula (Dufiex - SS White). The polystyrene resin (Cromitec) was manipulated and poured into the PVC rings until filling (10). After polymerization, the PVC tube and excess wax were removed with a Lecron spatula (Duflex-SSWhite).

-Occlusal surface wear

The occlusal surface of the teeth was worn in a rotary electric polisher (South Bay Technology), under water cooling, with silicon carbide sandpapers (Carborundum Abrasives- Ind. Bras.) in granulations of 180 and 320 until all the occlusal enamel was removed to expose a flat area in the superficial dentin. Polishing was performed using silicon carbide sandpapers in granulations of 600 and 1200.

Next, the samples were taken to a precision device (0.01 mm) specific for performing standard cavity preparations (10).

-Cavity preparation

Class I (occlusal) cavities were produced with the following dimensions: mesiodistal width of 6 mm, bucco-palatal width of 3 mm, and depth of 3 mm. The cavities were prepared using a rounded cylindrical diamond tip (FG 2143- KG Sorensen) at high rotation and under water cooling to obtain parallel walls and rounded angles (10). Every five preparations, the diamond tip was replaced. The cavities were finished with the same diamond tip at low rotation.

-Obtaining dental fragments and experimental groups

The roots were separated from the crowns through a cut parallel to the pulp chamber floor, made with a double-sided cutting disc (KG-Sorensen - Dentsply) at low rotation. The pulp tissue was removed with dentin spoons (Duflex-SSWhite) and the pulp chamber was cleaned with sodium bicarbonate and water blasts.

The impurities produced during the preparation of the dental fragments were removed with an ultrasonic bath (T-14 Thornton Impetch Eletr6nica Ltda.) for 12 minutes (11).

The crowns were randomized by a draw into six experimental groups with seven units each: 1) Single Bond adhesive associated with TPH composite resin; 2) Optibond Solo Plus and TPH adhesives; 3) Single Bond, Filtek Flow, and TPH; 4) Optibond Solo Plus, Filtek Flow, and TPH; 5) Vitremer, Single Bond, and TPH; and 6) Vitremer, Optibond Solo, and TPH.

-Restorative procedures

Group 1: 35% phosphoric acid gel (3M - ESPE) was applied inside and to the margins of the cavity and, after 15 seconds, it was washed with air/water blast for the same time. Excess water was removed with a damp cotton ball and the Single Bond bonding agent was applied abundantly to the entire surface of the preparation, aided by a microbrush (Microbrush Co. - USA) in two consecutive layers. After 30 seconds, a mild air blast was applied for three seconds and the adhesive was photoactivated (Degulux - Degussa) for 10 seconds. Therefore, the active tip of the device was protected and supported on the cavity margins (light intensity of 870 mW/cm2). Next, an oblique increment of TPH composite (color A3) with approximately 2 mm of thickness (recommended by the manufacturer) was inserted in the cavity aided by a Thompson spatula, taking care so that opposite walls were not bonded (12) and photoactivated for 20 seconds (recommended by the manufacturer). A new oblique increment was adapted on the opposite wall and photoactivated again. Two other oblique increments were adapted and respectively photoactivated to completely close the cavity, obtaining a flat surface in the same height of the wear previously performed.

Group 2: 37% phosphoric acid gel was applied inside the cavity and, after 15 seconds, it was washed with water blast for the same time. Excess water was removed with a dry cotton ball, according to the manufacturer’s recommendation, and the Optibond Solo Plus bonding agent was applied actively, aided by a microbrush, for 15 seconds. Then, a mild air blast was applied for three seconds and the adhesive was photoactivated for 20 seconds (recommended by the manufacturer). The composite resin restoration was performed as described for group 1.

Group 3: The dental structure was hybridized according to the recommendations of the manufacturer of the Single Bond system, as described for group 1. The Filtek Flow low-viscosity resin was applied carefully to the cavity floor and surrounding walls aided by a microbrush and photoactivated for 20 seconds, according to the manufacturer’s recommendation. Then, the composite resin restoration was completed as described for group 1.

Group 4: The Optibond Solo Plus bonding system was applied inside the cavity according to the manufacturer’s recommendation, as described for group 2. Then, the Filtek Flow low-viscosity composite resin was applied to the cavity floor aided by a brush and photoactivated for 20 seconds. The restoration was completed as described for group 1.

Group 5: Initially, the primer of the Vitremer system was applied to all cavity walls with the help of a brush. After applying a mild air blast, photoactivation was performed for 30 seconds. Vitremer powder and liquid were dispensed in a precision analytical scale at the ratio of 2.5: 1 in weight, meaning 5 mg of powder for 2 mg of liquid, and agglutinated with a 24 spatula (Duflex-SS White). The material was inserted carefully in the cavity floor aided by a Centrix syringe and extra-fine tip, and photoactivated for 40 seconds. Next, the Single Bond bonding system and the TPH composite were applied as described for group 1.

Group 6: Vitremer was applied as described for group 5. The Optibond Solo bonding system was applied inside the cavity according to the manufacturer’s recommendation, as described for group 2. Finally, composite resin restoration was performed as in group 1.

After completing the restorations, the samples were stored in a Fanem oven at 37°C and 100% relative humidity, for seven days. The dental slices restored were stabilized on acrylic plates, through the lingual surface, with previously heated godiva, and taken to the precision metallographic cutter. Using a high-concentration diamond disc, at the speed of 200 rpm (13) and under constant refrigeration with distilled water, two 0.8-mm-thick parallel cuts were made on the buccal aspect (14) and other cuts were made perpendicular to these two for obtaining slices in a parallelepiped shape. The slices obtained by cutting the dental crowns were used in the microtensile test. To assess the stresses produced by polymerization shrinkage on the walls surrounding the cavity preparation, a slice of each tooth was separated for evaluating the fracture interface through SEM analysis.

-Microtensile test

The specimens (fragments) were taken to an Instron universal machine (Instron Co.-England) and each end was placed on one of the surfaces of the specific device for the microtensile test, and the ends were stabilized with Super Bonder cyanoacrylate glue gel (Loctite- USA) and Zapit accelerator (DVA- Corona).

Loading was applied at a speed of 0.5 mm/min with a load cell of 5 kg until the specimen would rupture (13). The area of the interface where the fracture occurred was calculated and the strength values were obtained in Mega Pascal (MPa). These values were subjected to statistical analysis.

-Fracture interface analysis

The impurities deposited during the cutting of toothpicks were removed with an ultrasonic bath for 12 minutes. Then, the toothpicks were polished manually with wet silicon carbide sandpapers in granulations of 600 and 1200 and a felt disc associated with diamond paste. The samples were washed in running water for three minutes and then immersed in a hydrochloric acid solution (6M) for 30 seconds. Next, they were washed for three minutes and immersed in a sodium hypochlorite solution (1%) for 10 minutes. After washing under running water, the toothpicks were taken to the ultrasonic bath for 30 minutes.

To remove excess water, the toothpicks were stored in open Eppendorfs in a stove at 37°C for 12 hours. Metallization was performed in a metallizer (Desk II cold sputter/etch unit, Denton Vacuum Inc.) to evaluate the fracture interface. After obtaining a vacuum of 10-1 mmHg in an Argon atmosphere, a gold plate was bombarded by positives ions and the atoms produced by this bombarding of gold was deposited on the sample surface. To obtain a 200A surface layer, metallization was performed for three minutes. The samples were analyzed in SEM, operating at 14 kV, with a magnification of 500 times.

## Results

The data obtained in the microtensile strength test allowed performing Analysis of Variance (ANOVA) entirely at random. The ANOVA found f = 2.63 significant at 5%. However, the Levene test was applied, which indicated scale problems for the Response Variable. To correct this problem, the transformation of the data was indicated, elevating them to the power of 0.4. This transformation corrected the problem and a new Analysis of Variance was performed, which results are presented in Table 2.

**Table 2:**
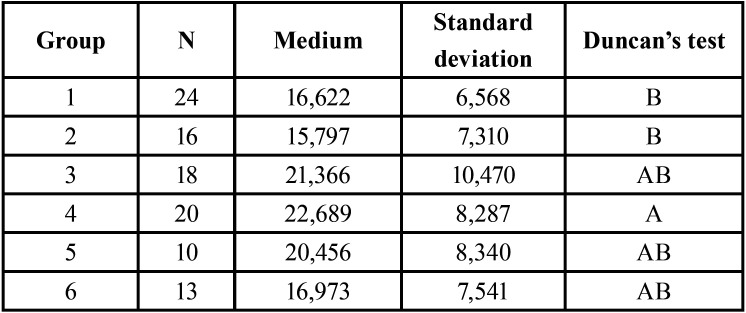
Result of Duncan’s test for the microtensile strength test.

This new Analysis of Variance indicated f = 2.55 significant at 5% of probability, with a coefficient of variation of 18.24%. To better elucidate these results, Duncan’s test was applied also at 5% of probability (Table 2).

The analysis of Table 2 showed that group 4 obtained the highest mean of microtensile strength, showing no statistically significant difference to groups 3, 5, and 6 and a statistically significant difference to groups 2 and 1. Moreover, there was no statistical difference between the other groups studied. Table 3 shows the classification of the fracture patterns analyzed after the microtensile test.

**Table 3:**
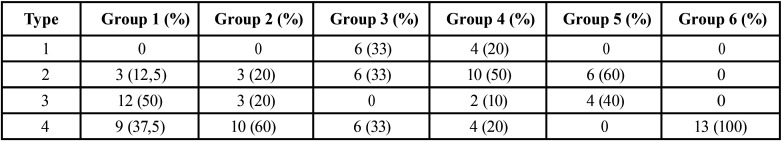
Classification of fracture patterns analyzed after the microtensile test. Type 1: adhesive; Type 2: mixed; Type 3: cohesive in dentin and Type 4: cohesive in the material.

## Discussion

Polymerization shrinkage is an intrinsic characteristic of composite resins that causes clinical inconveniences such as postoperative sensitivity, discoloration, and marginal cracks, and it may consequently lead to the development of recurrent caries (15). The clinical problems from polymerization shrinkage are accentuated when composites are used to restore posterior teeth (16). This is probably due to the high configuration factor (C-factor) found in these cavities. The C-factor is the ratio between the adhered and free surfaces of the cavity preparation, and the higher this ratio, the less freedom the material has to dissipate stresses during polymerization shrinkage (17).

Another aspect related to the clinical problems caused by composite resin shrinkage in posterior teeth is the volume of restorative material used. The higher the volume of composite inserted in the cavity, the higher the volumetric shrinkage of the restoration after polymerization. To minimize the deleterious effects of polymerization shrinkage, some clinical techniques may be used: loaded adhesives, which would provide a thicker adhesive interface, low- viscosity composite resins because the lowest load content of these materials, although resulting in a higher polymerization shrinkage, reduces the elasticity modulus and causes the stresses produced by composite shrinkage to dissipate rather than be transferred to the restorative interface and resin-modified glass ionomer cement for presenting a viscoelastic behavior that allows high stress dissipation before presenting plastic or definitive deformation (18-20).

The result of Duncan’s test showed that in the groups that associated the adhesive system and the flow composite resin (Filtek Flow/Optibond Solo Plus and Filtek Flow/Single Bond) obtained the highest means of microtensile strength, without a statistically significant difference to the groups that associated the adhesive system and the resin-modified glass ionomer (Vitremer/Single Bond and Vitremer/Optibond Solo Plus).

The result of the microtensile test shows that it is not only the volume of the base material that affects the concentration of stresses in the adhesive interface. The mechanical characteristics of the base material play an essential role in dissipating these stresses during the polymerization reaction of the composite resin. Low-viscosity hybrid composites present a lower load percentage, that is, from 20% to 25% lower than medium-viscosity composites; hence, these composites present a lower elasticity modulus (21). The elasticity modulus of the restorative composite has been indicated as an important factor in the development of stresses during the polymerization shrinkage of a dental composite, meaning that the higher the elasticity modulus, the more stress is produced during polymerization shrinkage, and these stresses are transmitted to the restorative interface (22-24). Thus, for presenting a lower elasticity modulus, the flow composite resin works as a layer of stress absorption during the polymerization shrinkage of the composite resin, decreasing stress in the restorative interface by up to 50% and helping to maintain marginal sealing (25-27). The relationship between modulus of elasticity and llinear polymerization shrinkage of low-viscosity resin composites when used as restorative material is a critical factor in contraction stress relief and marginal leakage. The results of this work corroborate the study by Xavier and collaborators in which they verified that conventional resin presented lower linear polymerization shrinkage and high flexural strength/modulus of elasticity (28).

The reduction of stress in the restorative interface through an ionomeric base material showed an intermediate behavior between the association of flow composite resin/adhesive system and adhesive system alone. This behavior may be credited to the polymer matrix of Vitremer, which also presents polymerization shrinkage, producing stresses in the adhesive interface, as well as to its higher elasticity modulus relative to the flow composite resin (29).

In this *in vitro* study, the main limitation related to the microtensile assay was the difficulty in obtaining the specimens, as it requires the use of specific equipment with greater time demand when compared to conventional assays. In the fracture test, investments on the loading protocol are necessary to standardize the speed of application of the test, as well as the shape and size of the tips for application of the load (30).

## Conclusions

The highest mean of microtensile strength was obtained when the volume of the restorative material decreased through the interposition between the material and the adhesive system of a base with low elasticity modulus.
